# Copper in Gynecological Diseases

**DOI:** 10.3390/ijms242417578

**Published:** 2023-12-17

**Authors:** Rocío A. Conforti, María B. Delsouc, Edith Zorychta, Carlos M. Telleria, Marilina Casais

**Affiliations:** 1 Facultad de Química, Bioquímica y Farmacia, Universidad Nacional de San Luis (UNSL), Instituto Multidisciplinario de Investigaciones Biológicas de San Luis (IMIBIO-SL-CONICET), San Luis CP D5700HHW, Argentina; ro.conforti64@gmail.com (R.A.C.); bdelsouc@gmail.com (M.B.D.); 2 Experimental Pathology Unit, Department of Pathology, Faculty of Medicine and Health Sciences, McGill University, 3775 University Street, Montreal, QC H3A 2B4, Canada; edith.zorychta@mcgill.ca; 3 Cancer Research Program, Research Institute, McGill University Health Centre, Montreal, QC H4A 3J1, Canada

**Keywords:** copper, gynecological diseases, Cu chelators, Cu ionophores, ovarian cancer, polycystic ovarian syndrome, cervical cancer, endometrial cancer, endometriosis

## Abstract

Copper (Cu) is an essential micronutrient for the correct development of eukaryotic organisms. This metal plays a key role in many cellular and physiological activities, including enzymatic activity, oxygen transport, and cell signaling. Although the redox activity of Cu is crucial for enzymatic reactions, this property also makes it potentially toxic when found at high levels. Due to this dual action of Cu, highly regulated mechanisms are necessary to prevent both the deficiency and the accumulation of this metal since its dyshomeostasis may favor the development of multiple diseases, such as Menkes’ and Wilson’s diseases, neurodegenerative diseases, diabetes mellitus, and cancer. As the relationship between Cu and cancer has been the most studied, we analyze how this metal can affect three fundamental processes for tumor progression: cell proliferation, angiogenesis, and metastasis. Gynecological diseases are characterized by high prevalence, morbidity, and mortality, depending on the case, and mainly include benign and malignant tumors. The cellular processes that promote their progression are affected by Cu, and the mechanisms that occur may be similar. We analyze the crosstalk between Cu deregulation and gynecological diseases, focusing on therapeutic strategies derived from this metal.

## 1. Introduction

Copper (Cu) is an essential micronutrient for the proper development of eukaryotic organisms [1]. Because it cannot be created or destroyed through metabolic processes, this metal must be acquired from external sources, primarily food and water. According to recommendations, adults should consume approximately 0.9 mg of Cu/day, and in conditions such as pregnancy and lactation, around 1.3 mg/day [2]. In general, the average intake of most people meets or exceeds this requirement since it is estimated that Cu ingested through food, water, and supplements ranges between 1.1 and 1.7 mg/day in adults [3], of which only 15% is retained in tissues: the rest is excreted through the bile and, to a lesser extent, through urine. This micronutrient is present at high concentrations in foods such as liver, crustaceans, red meat, milk, chocolate, seeds, fish, mushrooms, and nuts [4]. Cu is mainly accumulated in the liver, kidneys, brain, heart, muscles, and skeleton [5]. The serum concentration of Cu in healthy adults ranges between 70 and 110 mg/dL, where 70% is bound to its principal transporter, ceruloplasmin (Cp) [6,7].

As a vital trace element, Cu plays a key role in many cellular and physiological processes, such as enzyme activities, oxygen transport, and cell signaling. Being a catalytic cofactor of redox proteins, it is clear that Cu plays a crucial role in carrying out biological functions necessary for growth and development [8]. These functions are due to its two oxidation states: the reduced form (Cu^+^) and the oxidized form (Cu^2+^), which give it the ability to act as an electron recipient or donor. The extracellular environment contains mainly Cu^2+^, while inside the cells, the reduced form of Cu is found [9,10]. Cu^2+^ may regulate various growth factors and membrane receptors, while Cu^+^ is involved in intracellular regulation by affecting the activation state of membrane receptors or binding to transcription factors to alter gene expression [11]. Analysis of the human proteome has so far identified more than fifty Cu-binding proteins, of which some examples include Cu/Zn superoxide dismutase (SOD1), cytochrome C oxidase (CCO), Cp, lysyl oxidase (LOX), tyrosinase, and dopamine-β-hydroxylase (DβH), among others (Table 1). The main functions of Cu involve oxidation-reduction reactions that ultimately produce free oxygen radicals. For this reason, free cellular Cu concentrations must be maintained at low levels [8]. Given its essential role in cellular physiology, it is important to understand the mechanisms related to Cu metabolism in biological systems.

### 1.1. Copper Metabolism

**Figure 1:**
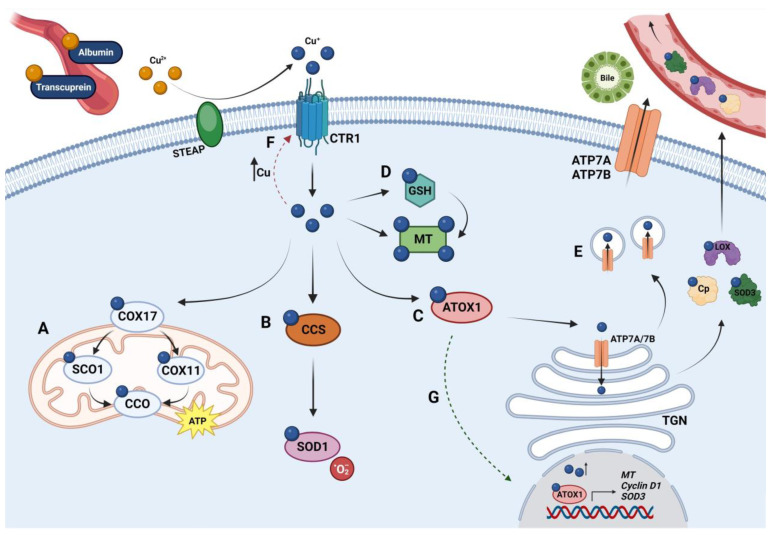
Schematic diagram of copper metabolism in mammals. After intestinal absorption, Cu travels through the portal vein bound to soluble proteins, such as albumin and transcuprein. On the surface of mammalian cells, metalloreductases such as STEAP 2, 3, and 4 reduce Cu^2+^ ions to Cu^+^ so that cells can absorb Cu through CTR1. (A) In the mitochondrial intermembrane space, COX17 is responsible for delivering Cu^+^ to either SCO1 or COX11 to contribute to the correct assembly of CCO, which utilizes Cu for energy production through oxidative phosphorylation. (B) CCS chaperone transfers Cu^+^ to SOD1, which is critical in the defense against oxidative stress because it catalyzes the degradation of superoxide radicals. (C) ATOX1 is responsible for providing Cu to the ATPases (ATP7A and ATP7B) that are principally located in the trans-Golgi network (TGN). ATPases pump Cu^+^ from the cytosol into the lumen of the TGN to promote the synthesis of cuproenzymes, such as Cp, LOX, and SOD3, which are secreted out of the cells to mediate the Cu transport through the circulatory system. (D) Since free Cu ions have the potential to generate reactive oxygen species, excess intracellular Cu^+^ is sequestered mainly by glutathione (GSH) and metallothioneins (MTs) that uptake Cu for storage. GSH can also deliver Cu to MTs. (E) When the cytoplasmic Cu concentration increases, ATP7A and ATP7B move within endocytic vesicles toward the plasma membrane to transfer excess Cu into the bloodstream. ATP7A is expressed in many tissues except in the liver, where it is replaced by ATP7B. In hepatocytes, ATP7B ensures the movement of Cu through the canalicular membrane for its subsequent elimination through the bile. (F) The concentration of mammalian CTR1 at the plasma membrane is negatively regulated in response to elevated Cu levels (red dotted arrow), with CTR1 being removed from the cell surface. (G) ATOX1 can carry Cu into the cell nucleus and act as a transcription factor for the expression of genes encoding cyclin D1 and SOD3 (green dotted arrow). High concentrations of cellular Cu may also stimulate the transcription of MT genes. Created with BioRender.com (accessed on 11 November 2023).

In mammals, Cu absorption, distribution, storage, and excretion take place at both systemic and cellular levels. During the last 20 years, the mechanisms related to these processes have been widely studied [12]. A schematic diagram of Cu metabolism in mammals is shown in Figure 1. Cu homeostasis depends mainly on the precise regulation of these processes by organ systems and individual cells. Studying the various alterations that may cause Cu dyshomeostasis is one of the most attractive focuses in Cu research at the present time.

#### 1.1.1. Copper Uptake

Copper is acquired mainly from food and water and is absorbed through the intestinal epithelium to reach the liver through the portal vein [10]. In the digestive tract, Cu^2+^ can be incorporated by epithelial cells through the action of divalent metal transporter 1 (DMT1); however, specific deletion of *Dmt1* in enterocytes does not prevent intestinal absorption of Cu [13], indicating that other mechanisms of transport are also in place. The reduction of Cu^2+^ by metalloreductases such as DCYTB and STEAP 2, 3, and 4 on the surface of mammalian cells [14,15] allows Cu^+^ ions to then be incorporated by CTR1 (copper transporter 1, encoded in humans by the *Slc31a1* gene). CTR1 is a high-affinity Cu importer belonging to the SLC31 family, and it plays a fundamental role in Cu homeostasis, being the main pathway of Cu^+^ incorporation into cells [1]. It is located on the apical membrane of enterocytes [16], but it can also be found on the basolateral membrane and within intracellular organelles [17]. CTR1 is a homotrimeric protein that forms a pore in the membrane, where each monomer displays an extracellular N-terminal domain for Cu binding [18]. It also has three transmembrane domains (TMDs): TMD1 and TMD2 interact with Cu, and TMD3 is essential for CTR1 oligomerization. The cytoplasmic C-terminus allows intracellular delivery of Cu by undergoing conformational changes upon metal binding [19]. Enterocyte-specific *Ctr1* knockout mice experience severe Cu deficiency in peripheral tissues, cardiac hypertrophy, liver iron overload, and severe growth and viability defects [20], while systemic inactivation of CTR1 leads to embryonic death [21], confirming the importance of CTR1 in Cu uptake and normal cellular function.

#### 1.1.2. Copper Distribution

The Cu-transporting ATPase α (ATP7A) in intestinal epithelial cells is the essential protein transporting Cu from the intestine to the rest of the body. ATP7A is expressed in many tissues except the liver, where it is replaced by its paralog, Cu-transporting ATPase β (ATP7B) [22]. ATP7B is mainly expressed in the liver, kidney, heart, brain, placenta, and lung [22]. In the placenta and blood–brain barrier, ATP7A ensures sufficient amounts of Cu for proper development of the fetus and brain [23]. After absorption into the enterocyte, ATP7A secretes Cu into the portal circulation, where it binds to soluble chaperones, including albumin, transcuprein, and macroglobulins [24,25,26]. Upon reaching the liver, Cu enters hepatocytes through CTR1, and the liver becomes the main depot of Cu in the body, distributing it to peripheral organs through the bloodstream or excreting it through the bile [27]. Within the cytoplasm, Cu trafficking is tightly coordinated by high-affinity Cu chaperones that deliver Cu to specific proteins and metallothioneins (MTs) that bind Cu for storage [22,24,28,29]. The major Cu chaperones include cytochrome C oxidase (CCO), Cu chaperone for SOD (CCS), and antioxidant chaperone 1 (ATOX1).

Cytochrome C oxidase utilizes Cu for mitochondrial function and oxidative phosphorylation (Figure 1A). CCO consists of two subunits, COX1 and COX2, which bind Cu at conserved sites [30]. The Cu chaperone COX17, located in the mitochondrial intermembrane space (IMS), transports Cu from the cytosol to the IMS to contribute to the correct assembly of CCO [28]. In the IMS, COX17 delivers Cu^+^ to SCO1 (synthesis of cytochrome C oxidase 1) for transfer to the COX2 subunit or COX11 for delivery to the COX1 subunit [31,32]. Other participants could be involved in Cu trafficking to mitochondria, such as COX19 and a non-protein, anionic copper ligand [32]. Mitochondria provide the main intracellular reservoir of Cu, which is essential for their energy production through oxidative phosphorylation [8,32]. Within enterocytes and other cells, CCS delivers Cu to the SOD1 enzyme to scavenge free radicals (Figure 1B). A recent study suggested that CCS first acquires Cu from CTR1 and then delivers it to SOD1 by forming a CTR1-CCS-SOD1 complex that can be dissociated upon SOD1 activation [33]. CCS expression is regulated by cellular Cu content because when Cu levels decrease, CCS increases, while when Cu content increases, this chaperone is degraded [34]. The SOD family of proteins is critical in the defense against oxidative stress because they catalyze the degradation of superoxide radicals into hydrogen peroxide and oxygen [35]. There are several isoforms of SOD, of which SOD1 (intracellular dimeric) and SOD3 (extracellular tetrameric) contain Cu, whereas SOD2 is a mitochondrial enzyme that contains Mn. In addition, ATOX1 is responsible for transferring Cu to ATP7A and ATP7B, which are membrane pumps characterized by eight TMDs, including multiple Cu binding sites located mainly on TMD6, TMD7, and TMD8 (Figure 1C) [36,37]. These ATPases are located in the trans-Golgi network (TGN), in endocytic vesicles, or in the plasma membrane, pumping Cu^+^ from ATOX1 to the other side of the membrane [38]. The central role of ATOX1 is reflected in the perinatal death of *Atox1* knockout mice due to the altered Cu balance [39].

Since free Cu ions have the potential to generate reactive oxygen species (ROS) in cells, excess intracellular Cu^+^ must be sequestered by molecules such as MTs and glutathione (GSH) (Figure 1D). MTs are a family of low-molecular-weight proteins capable of binding excess Cu^+^ ions through thiol groups [29]. In humans, four distinct MTs are known: MT1, MT2, MT3, and MT4. MT1 and MT2 are widely expressed throughout the body, while MT3 and MT4 are principally expressed in the central nervous system [29]. Glutathione is a tripeptide containing glutamate, cysteine, and glycine residues that is also capable of buffering excess Cu. It is probably the first acceptor of Cu as soon as it enters the cell [40,41]. Millimolar cytoplasmic GSH concentrations are estimated to markedly exceed Cu levels [42]. This fact enables GSH to act as a cytosolic Cu buffer that prevents the rise of free Cu ions and drives CTR1-mediated Cu influx by maintaining a negative concentration gradient at the plasma membrane [40]. GSH and other molecules with thiol groups, together with the enzyme, glutaredoxin 1, may generate a reducing environment conducive to the redox regulation of ATP7A and ATP7B, modulating the binding of Cu to cysteine residues, being fundamental for the export of the metal [41].

#### 1.1.3. Copper Excretion

After being stored, Cu can be released into the bloodstream for subsequent distribution to specific tissues and organs [12,24]. This occurs through several pathways, where ATP7A and ATP7B are the central players. These ATPases have a dual role in the cell; first, they have a biosynthetic function because they promote the synthesis of enzymes loaded with Cu (cuproenzymes) in the TGN, such as Cp, LOX, and tyrosinase, which are then secreted out of the cells (Figure 1C) [23]. Cp is the main transport medium for Cu in the circulatory system; therefore, the abundance of Cp in plasma may serve as a biological marker of systemic concentration of this metal [43,44]. In addition, ATP7A and ATP7B have a homeostatic function because when the cellular concentration of Cu increases, they move within endocytic vesicles toward the plasma membrane to transfer excess Cu out of the cell (Figure 1E) [45]. In hepatocytes, ATP7B ensures the movement of Cu through the canalicular membrane for its subsequent elimination through the bile so that any overload is excreted through the digestive tract [38]. Although biliary excretion is the main form of endogenous Cu excretion, there are other routes for Cu elimination, such as urine, sweat, and menstruation [24].

### 1.2. Copper Homeostasis

Although the redox activity of Cu is essential for enzymatic reactions, this property also makes it potentially toxic at high levels [12]. During the change between Cu^+^ and Cu^2+^ states, electron transfer results in the generation of ROS, including superoxide anion (O^2−^), nitric oxide (NO), hydroxyl radical (OH^−^), and hydrogen peroxide (H_2_O_2_), via the Fenton reaction [46]. ROS can attack bio-membranes, destabilizing their structure and affecting their cellular functions, and can also oxidize proteins and denature DNA and RNA, altering the repair mechanisms of these nucleic acids [47]. All of these changes may contribute to the development of cancer, neurodegenerative diseases, and cellular aging [48]. In contrast, a deficiency in Cu can lead to alterations in energy levels, glucose and cholesterol metabolism, and immune cell function, increasing the risks of infections and cardiovascular disorders [44,49,50]. The activities of SOD1, Cp, catalase, and glutathione peroxidase, as well as MT and GSH, are also compromised by an imbalance in the levels of Cu [44]. The dual roles of Cu as an essential and toxic element require specific regulatory mechanisms to prevent both deficiency and accumulation since dyshomeostasis can promote the development of multiple diseases, affecting liver function, lipid metabolism, the central nervous system, and resistance to chemotherapy, among others [51].

Copper homeostasis is highly regulated by transcriptional control and selective transport mechanisms [47]. High levels of cellular Cu negatively regulate the concentration of mammalian CTR1 at the plasma membrane, which trigger CTR1 removal via endocytosis-dependent internalization or degradation (Figure 1F) [52]. In contrast, when Cu concentration is reduced, internalized CTR1 returns to the plasma membrane [53]. In vitro studies have shown that transcription of the *Ctr1* gene is regulated by the transcription factor Sp1 (Specificity protein 1) in a Cu-dependent manner, where overload produces a negative regulation of *Ctr1* [54]. In vivo, mice fed a Cu-deficient diet had increased CTR1 expression in the intestine [55]. CTR1 function can also be regulated via the generation of a truncated protein (tCTR1) through the removal of its high-affinity Cu-binding domain [56]. tCTR1 is produced within endosomal compartments, has lower uptake activity than CTR1, and requires interactions with CTR2 (copper transporter 2), which is the only other SLC31 family protein in mammals. Initially, CTR2 was proposed as a low-affinity Cu transporter; however, it is currently believed that CTR2 has lost the ability to transport Cu and that its primary role is to produce tCTR1 [57].

Another protein involved in Cu homeostasis is the ATOX1 chaperone, which can act as a transcription factor stimulated by Cu, translocating to the nucleus to bind to promoters of genes that encode cyclin D1, the organizer of the nicotinamide adenine dinucleotide phosphate (NADPH) oxidase p47phox, and SOD3 (Figure 1G) [58,59,60]. It has also been reported that high concentrations of cellular Cu can improve MT gene transcription, mediated by metal-regulatory transcription factor 1 (MTF1) and nuclear factor erythroid 2-related factor 2 (Nrf2) [61,62].

Regulating the localization and function of ATP7A and ATP7B is essential in controlling Cu export from the cell [23]. At physiological levels of Cu, these transporters pump Cu from the cytosol into the lumen of the TGN to load Cu^+^ into cuproenzymes, which mediate the transport of Cu through the circulatory system [7]. When intracellular Cu increases, ATPases move to the post-Golgi vesicular compartments, which are loaded with Cu, and release this metal into the extracellular medium after fusion with the plasma membrane [38,45]. After Cu levels are restored to physiological levels, ATP7A and ATP7B are transported back to the TGN through the action of several protein complexes, such as AP-1, Arp2/3, WASH, and COMMD/CCDC22/CCDC93 [63].

### 1.3. Copper and Pathogenesis

The participation of Cu in both the development and progression of diseases has been documented in numerous reports that show an alteration of Cu homeostasis with aberrant levels of this metal. Mutations in the genes encoding ATP7A and ATP7B cause inherited disorders of Cu metabolism, known as Menkes’ disease and Wilson’s disease, respectively [64,65]. Menkes’ disease is an X-linked recessive disorder, fatal to male infants, in which the dysfunction of ATP7A leads to reduced Cu availability in tissues, causing growth retardation, hypotonia, kinky, brittle hair (pili torti), deterioration of the nervous system, and severe intellectual disability [64]. Wilson’s disease is an autosomal recessive disorder characterized by a profound accumulation of Cu, primarily in the liver, brain, and kidneys, due to mutations in the ATP7B gene that impair the ability to excrete Cu into the bile. This triggers hepatic and neuropsychiatric symptoms in these patients [65,66].

In addition to the genetic disorders described above, Cu dyshomeostasis has been associated with a large number of diseases, namely neurodegenerative disorders, such as Alzheimer’s, Parkinson’s, and Huntington’s diseases, and amyotrophic lateral sclerosis [67,68], as well as atherosclerosis [69], diabetes mellitus [70], and cancer [10,71,72]. Recent studies have demonstrated a strong correlation between Cu and three fundamental processes for tumor progression: cell proliferation, angiogenesis, and metastasis [71,72]. Cu also has a role in oxidative stress and chronic inflammation, which promote cell transformation [35,73]. Furthermore, gene expression analysis has revealed multiple alterations in Cu-sensitive or Cu-binding proteins [74], which indicate a relationship between Cu dyshomeostasis and cancer pathogenesis. Therefore, it has been proposed that an important risk factor for carcinogenesis could be elevated levels of Cu in tissues or serum [47,75]. Preclinical studies demonstrated that daily administration of CuSO_4_ through drinking water significantly increased tumor growth in a murine model of breast cancer [76]. In conjunction with these results, elevated Cu levels in serum and malignant tissues have been documented in different human cancers, including breast, gastrointestinal, and gynecological malignancies [77,78,79,80]. While Cu elevation in cancer cells may be involved in carcinogenesis, it could also be a feature of the cancer phenotype for two main reasons. Tumors, especially fast-growing ones, have greater metabolic demands than healthy tissues that do not divide [47]. As Cu is a cofactor for multiple enzymes in cellular energy metabolism, such as CCO, and in antioxidant defenses, such as SOD [47,77], the demand for Cu could increase in cancer cells. Second, in tissues undergoing hypoxia, upregulation of CTR1 has been observed [81]. Hypoxia-inducible factor 1-alpha (HIF-1α) may activate the transcription of genes related to Cu metabolism (e.g., those that control CTR1), contributing to higher Cu levels in hypoxic tumor cells [82].

Due to the popularity of copper intrauterine devices (Cu IUDs) as a contraceptive method interfering with fertilization and/or implantation [83], it would be interesting to evaluate whether their use could modify serum Cu levels. In a recent review [84], eight of twelve studies analyzed found that Cu IUDs would not change the serum concentrations of this metal. Although the in situ release of Cu ions is very low, research is limited, and there is no clear evidence. Since Cu IUD users generally experience abnormal uterine bleeding or abdominal pain and have not shown clinical signs of toxicity, it is believed that Cu homeostasis mechanisms may be sufficient to prevent the accumulation of this metal. In an animal study, Wistar rats were implanted with Cu IUDs of different doses, with no toxic effects evident [85]. Because they were exposed to much higher Cu levels than those used in humans, the results are reassuring about the Cu IUDs’ safety; however, more research is needed.

Although Cu is involved in a spectrum of diseases, its role in cancer has been the most studied, permitting an analysis of how this metal can affect different cellular processes related to tumor progression. This will be described in the following subsections, and subsequently, our focus will be to evaluate the crosstalk between Cu deregulation and gynecological diseases, which mainly include benign and malignant tumors. The mechanisms that occur in both types of tumors may be similar, where the starting point is abnormal cell proliferation [86]. Finally, we will focus on new Cu-based therapeutic strategies, especially for those gynecological diseases with high prevalence, morbidity, and mortality that do not respond adequately to other treatments.

#### 1.3.1. Copper and Cell Proliferation

Cuproplasia is defined as Cu-dependent cell growth and proliferation that can lead to neoplasia and hyperplasia [10]. This process is related to mitochondrial respiration, redox signaling, autophagy, antioxidant defense, and kinase signaling and may involve enzymatic and non-enzymatic Cu activities [10]. It has been observed that CCS can promote carcinogenesis. For example, in patients with breast cancer, the levels of CCS were increased along with the ability of CCS to promote proliferation through the MAPK/ERK pathway [87]. It was also shown that a specific inhibitor of CCS and ATOX1 reduced cancer cell proliferation and tumor growth [88]. Another emerging concept, metalloallostery, has expanded knowledge about the contributions of Cu to cellular signaling events since it proposes a new paradigm in which the dynamic binding of Cu occurs at sites other than the active sites of proteins to regulate them [89]. In the context of positive metalloallostery, Cu directly binds to MEK1 and MEK2 kinases and enhances their ability to phosphorylate ERK1 and ERK2 in a dose-dependent manner, stimulating the RAF–MEK–ERK signaling cascade, and ultimately, further promoting tumor proliferation [90]; this makes Cu an attractive target as this signaling cascade is one of the best-defined axes that promote cell proliferation and it is abnormal in most human cancers.

Autophagy is a cellular degradation process that plays an essential role in the development and differentiation of cells, constituting a means to cope with intracellular and environmental stress and potentially promoting tumor progression [91]. Recent studies have shown that increased intracellular Cu promotes the growth and survival of cancer cells by activating autophagy, stimulating the autophagic kinases ULK1 and ULK2 [91,92].

#### 1.3.2. Copper and Angiogenesis

One of the main processes involved in tumor growth is angiogenesis, where vascular endothelial cells migrate, proliferate, and differentiate to create a network of new blood vessels extending from surrounding vessels into the expanding tumor [93]. Angiogenesis is regulated by angiogenic-stimulating factors (angiogenin, vascular endothelial growth factor [VEGF], fibroblast growth factor [FGF], transforming growth factor beta [TGF-β]), and interleukins (IL-1, IL-6, IL-8), as well as through inhibitors (angiostatin and endostatin). The role of Cu as a pro-angiogenic metal was first proposed in 1980, with the discovery that Cu salts induce endothelial cell migration, an early step in angiogenesis [94]. Cu may be involved in the entire angiogenic signaling cascade, promoting the growth and mobility of vascular endothelial cells, regulating the synthesis and secretion of the main pro-angiogenic mediators (VEGF and FGF), and directly binding to angiogenin to modulate its affinity for endothelial cells [95]. Cu-dependent activation of HIF-1α transcriptional activity requires interaction with CCS, inducing the expression of pro-angiogenic genes [96]. Cu chelation has been shown to block HIF-1α-mediated VEGF expression [96,97] and to suppress the transcriptional activity of nuclear factor kappa B (NF-κB), thereby inhibiting the expression of FGF, VEGF, IL-1, IL-8, and IL-6 [98,99]. Overexpression of Cu-dependent SOD1 markedly increases VEGF production, while a reduction in SOD1 activity induces vascular abnormalities and impairs angiogenesis [100]. Additionally, Cu ions increase NO production, an inducer of vascular dilation, by activating endothelial nitric oxide synthase [95].

Copper transporters and chaperones also participate in angiogenesis. Upon stimulation by VEGF, cysteine 189 of the cytoplasmic C-terminal domain of CTR1 is sulfenylated, leading to the formation of a disulfide bond between CTR1-VEGFR2 and its co-internalization into early endosomes, promoting angiogenesis via VEGFR2 [101]. Indeed, silencing CTR1 expression in Cu-treated endothelial cells inhibits tube formation and reduces VEGF expression [102]. Regarding ATOX1, since it can act as a transcription factor for NADPH oxidase, it causes inflammatory neovascularization [58]. ATOX1 may also stimulate cyclin D1 in a Cu-dependent manner [59], potentially contributing to cancer cell proliferation and angiogenesis. In this regard, depletion of ATOX1 inhibits vascular smooth muscle cell migration stimulated by platelet-derived growth factor (PDGF), supporting a role for ATOX1 in vascular remodeling and tumor angiogenesis [103]. There may also be a role for ATP7A, as it can limit the degradation of VEGFR2, thereby promoting angiogenesis [104]. In summary, blocking Cu-dependent angiogenesis is an interesting strategy that can be further explored to inhibit tumor growth.

#### 1.3.3. Copper and Metastasis

Processes such as the development of pre-metastatic niches, escape from immune defenses, and angiogenesis will advance and sustain cancer progression. Cu and its binding proteins are involved in the metastatic spread of tumors [105], playing a critical role in the metastatic cascade, both within cells and in the tumor microenvironment. Cu participates in the epithelial–mesenchymal transition (EMT), an early step of metastasis, conferring migratory and invasive capabilities to the cancer cells [71,106,107]. In EMT, molecular reprogramming occurs, deactivating the expression of genes that encode epithelial markers, such as E-cadherin and occludin, and activating mesenchymal genes, such as N-cadherin and vimentin, which are targets of several transcription factors (Snail, Twist, Slug) [106]. The participation of Cu in the remodeling of the extracellular matrix (ECM) and the establishment of a pre-metastatic niche occurs mainly through the activity of LOX and Cu-dependent LOX-like (LOXL) proteins [108,109], which catalyze the cross-linking of collagen and elastin in the ECM. When LOX is active, it stimulates transcription via Twist to promote EMT in the tumor environment [110], and increased expression of LOXL2 correlates with metastasis and poor survival in breast cancer patients [111]. Both angiogenesis and metastasis were suppressed with LOX inhibitors during carcinogenesis examined in vivo, and the decrease in LOX expression inhibited cell migration and neovascular formation in tumor endothelial cells [112].

Adaptation to microenvironmental stressors such as hypoxia is an early characteristic of growing tumors, where HIF-1α plays a key role [113]. Cu and CCS activate HIF-1α by regulating binding to hypoxia-response elements (HREs), promoting the transcription of the target genes involved in EMT [96]. Indeed, Cu depletion in a tumor cell line inhibited the cellular characteristics of hypoxia-induced EMT by downregulating the expression of vimentin and fibronectin genes, which are under the control of the HIF1-α/Snail/Twist signaling pathway, and Cu depletion also inhibited angiogenesis in a mouse model [107]. HIF-1α also induces the expression of LOX, which promotes the synthesis of the HIF-1α protein upon activation of the PI3K/Akt pathway. Therefore, the synergistic action and regulation of both proteins results in the promotion of tumor progression [113,114]. The mediator of the cell motility 1 (MEMO1) protein was identified as a pro-metastatic mediator in breast cancer, where it acts as a Cu-dependent redox protein that promotes a more oxidized intracellular environment through the production of ROS [115]. MEMO1 is thought to have a metal-binding pocket similar to that of metal-dependent redox enzymes, where Cu can be coordinated to favor ROS production [115].

## 2. Copper in Gynecological Diseases

Over the years, investigating the role of Cu has gained increasing importance, and researchers have joined forces in trying to understand its action. As we previously described, Cu is a crucial element involved in each step of cancer development, from tumorigenesis to metastasis, and there is a large amount of research on the role of Cu in various types of cancer. However, to date, there are very few studies on the specific role of Cu in gynecological diseases [73,77,116]. These diseases mainly include benign and malignant tumors and endocrine diseases [117]. We will evaluate the impact of Cu dyshomeostasis in these diseases, focusing on therapeutic strategies based on altering the role of Cu.

### 2.1. Ovarian Diseases

#### 2.1.1. Ovarian Cancer

Gynecological cancers include all cancers that affect the female reproductive organs, including endometrial cancer, cervical cancer, ovarian cancer (OC), fallopian tube cancer, vaginal cancer, and vulvar cancer. Among the different gynecological cancers, OC is the most lethal worldwide [118,119]. More than 20 microscopically distinct types of OC can be identified, which are mainly classified into three groups: (1) epithelial cancers, (2) germ cell tumors, and (3) specialized stromal cell cancers [117]. Although significant progress has been made in early detection and treatment, OC is usually detected at a late stage and has a poor prognosis [120]. The overall 5-year survival rate for epithelial OC (EOC), which comprises about 90% of ovarian malignancies, is approximately 30% [119]. In addition to genetic and reproductive risk factors, it has been postulated that chronic inflammation, oxidative stress, and damage caused by free radicals to epithelial cells play a fundamental role in ovarian carcinogenesis [121]. EOC cells form spheroids to avoid immune detection and to resist cell death, where communication between these cells and the peritoneal ecosystem plays a crucial role in the progression and dissemination of the disease [122].

Elevated Cu levels have been reported in the serum of patients with OC [123,124,125], and it is also elevated in OC tumors [80], possibly due to alterations in trace elements with a reduced catabolism or an increase in the neoplastic synthesis of Cp, since elevated levels of both Cu and Cp have been found in patients with OC [123]. A meta-analysis demonstrated not only an increase in circulating Cu concentration but also a decrease in Zn levels in patients diagnosed with OC [125]. In another study, Cu levels were found to be elevated in patients with OC or endometrioma compared to the control groups [124]. However, a meta-analysis showed that using any type of IUD, including Cu IUDs, was associated with a lower incidence of OC [126]. A recent bioinformatics study demonstrated that analyzing the prognostic signature of Cu metabolism-related genes (CMRGs) could provide a useful predictive biomarker and a potential therapeutic target for patients with OC [127]. Additionally, the study showed that CMRGs help define the immune environment, which could serve to identify specific patient subgroups to receive specialized treatment.

The first-line treatments for OC are cytoreductive surgery and platinum-based chemotherapy [128]. Although the response rate is high, most patients typically experience relapses within 2 to 3 years [128]. At first relapse, 25% of patients have platinum resistance or refractory disease with a poor prognosis [129,130]. Numerous studies have identified the transport mechanisms of platinum-containing drugs [131], where it has been observed that many of the proteins involved also participate in Cu homeostasis. Reduced CTR1 expression has been related to cisplatin (CDDP) resistance in patients with OC [132], and higher CTR1 expression has been associated with a better response to CDDP treatment and favorable overall survival [133]. However, ATP7A and ATP7B are necessary to confer resistance to CDDP, carboplatin, and oxaliplatin in OC cell lines [131,134]. ATP7A- and ATP7B-dependent chemoresistance is linked to the impaired accumulation of CDDP in the nucleus and, consequently, the decreased formation of platinum-DNA adducts [131]. Other studies have proposed that CDDP binds to the Cu binding site of ATOX1 and is then transferred to ATP7B, promoting CDDP resistance [135,136]. However, the knockout of ATOX1 did not affect the acquisition of resistance to CDDP, indicating that other mechanisms are involved [137].

#### 2.1.2. Polycystic Ovary Syndrome

Polycystic ovary syndrome (PCOS) is an endocrine and metabolic disorder that occurs in approximately 6% to 20% of women of reproductive age and is a leading cause of infertility [138]. According to a large community-based cohort study, 72% of PCOS patients were infertile compared to 16% of the control group [139]. This disease is characterized by menstrual disorders, polycystic ovaries, and phenotypes related to hyperandrogenism, such as acne, alopecia, and hirsutism [140,141], in addition to a higher risk of spontaneous abortion and pregnancy-related complications [142]. PCOS is associated with obesity, dyslipidemia, insulin resistance (IR), type 2 diabetes mellitus, cardiovascular diseases, and endometrial cancer [143,144,145,146,147]. Regarding the etiology of PCOS, increasing evidence suggests that it could be a multifactorial and polygenic disorder with considerable epigenetic and environmental implications, including dietary and lifestyle factors [148,149,150].

The role of Cu in PCOS is complex and may vary with the phenotype. Several studies have found elevated Cu levels in patients with PCOS [151,152,153,154,155,156,157,158,159,160], while others have found no differences from the control group [161,162,163]. Considering metabolic factors, a significant increase in serum Cu levels was found in both obese and non-obese patients with PCOS compared to healthy subjects [153], and this increase was linked to IR [152,164]. Consequently, controlling Cu in these patients has been recommended as a potential strategy to lower oxidative stress and IR that could be caused by this metal and to minimize long-term metabolic complications [154]. Another study confirmed that patients with PCOS and IR had higher Cu levels than those without IR; however, Cu levels were lower in patients with PCOS than in the control group [165], similar to another work [166]. When Cu levels were measured in the follicular fluid, concentrations were higher in patients with PCOS than in controls [155], and this increase could negatively affect the development of follicles and be related to anomalies in steroidogenesis. Consistently, other investigators found that dietary intake of Cu was positively correlated with the risk of PCOS, and that this metal altered ovarian steroidogenesis, affecting ovarian follicle development [167], promoting premature follicular atresia, and inhibiting follicular maturation and the formation of multiple follicles.

### 2.2. Uterine Diseases

#### 2.2.1. Uterine Cervix Cancer

Uterine cervix cancer or cervical cancer (CC) is the fourth most common cancer in women worldwide, particularly in developing countries, making it a significant health problem [118]. High-risk human papillomavirus (HPV) infection is considered responsible for more than 90% of CC cases [168], so the prevalence varies depending on the prevalence of HPV infection [169]. Immunization against this virus can help prevent CC, and HPV testing is essential for early CC detection [170,171]. The overall 5-year survival rate is close to 66%; however, as treatment options are limited, patients with metastatic or recurrent disease have a lower survival rate [120]. Aside from HPV infection, some of the most relevant factors for the pathogenesis of CC are inflammation of the epithelium, elevated levels of lipid peroxides, reduced levels of non-enzymatic antioxidants, and altered activities of antioxidant enzymes [172]. One study found that Cu IUD use is not associated with cervical neoplasia [173], and patients have a lower risk of high-grade cervical lesions than oral contraceptive users [174].

High levels of Cu have been found in most studies in patients with CC. An early investigation observed a higher tissue concentration of Cu and a higher Cu/Zn ratio in patients with CC, along with a decrease in Zn levels compared to the control group [175], these results being confirmed in a later study [176]. A meta-analysis recently documented the association between increased serum Cu levels and CC risk [177], and subsequently, this association was confirmed for cervical and endometrial cancers as well as OC. CC patients had the highest Cu concentrations [178], and this increase was positively correlated with the stages of the disease, while Cu decreased after different treatments (surgery, chemotherapy, radiotherapy, or a combination of both) [179]. This result differs from a study where the authors observed that increased serum Cu levels were not modified after chemoradiotherapy in patients with CC [180]. In summary, Cu is indicated as a possible risk factor associated with CC that could be useful to monitor this type of cancer and potentially to control the progress of the disease [177,178,179].

#### 2.2.2. Endometrial Cancer

Uterine cancer or endometrial cancer (EC) is the fifteenth most common cancer in general and the sixth most common cancer in women [118]. Risk factors for the development of EC are obesity, high levels of estrogen, low levels of progesterone, PCOS, IR, diabetes, and estrogen-secreting ovarian tumors [181]. Most patients with early-stage disease have a good prognosis; however, the 5-year overall survival rate for advanced EC is 47% to 69% in stage III and 15% to 17% in stage IV [182]. Specific serum markers have not been established for clinical use in patients with EC. Regarding Cu, there is limited research on EC. A recent analysis evaluated the serum concentrations of Cu and Zn in patients with EC, finding lower levels of these metals compared to the control group [183]. In turn, patients with a greater degree of myometrial invasion had lower Cu levels than those with less myometrial invasion. In contrast, one study found elevated Cu levels without alteration in Zn levels [184], and others reported no changes in tissue Cu in patients with EC [80] or serum Cu in patients with OC and EC [185]. Other investigators found higher mean Cu levels in the serum of EC patients, but the results were not statistically significant. However, the authors observed that the menopausal status and body mass index of the patients were risk factors for EC, which may be affected by Cu concentrations [186]. Furthermore, in a review, the authors found that Cu IUD use could reduce the risk of EC, but the mechanism of action is unclear [187]. It is evident that the results obtained over the years have contradicted each other; therefore, more studies evaluating Cu levels are required to determine the possible clinical relevance in patients with EC.

#### 2.2.3. ‘Benign’ Diseases

Benign neoplasms have received less attention than malignant tumors, probably due to the biased view that ‘malignant’ is life-threatening and ‘benign’ has little effect. While this may be true, benign tumors can put pressure on vital organs, disrupt hormonal balance, and become malignant over time. Although both types of tumors have marked differences (for example, the ability to metastasize), they can be very similar at a mechanistic level, starting from abnormal cell proliferation [86].

Benign uterine diseases are common gynecological disorders in women of reproductive age, and this category includes endometrial polyps [188], uterine leiomyomas [189], and endometriosis [190], among others. Symptoms range from dysmenorrhea and irregular uterine bleeding to the risk of infertility. Associated factors are age, diet, lifestyle, pregnancy, abortion, and hormone use [188,189,190,191]. Endometrial polyps are common endothelial tumors that cause abnormal uterine bleeding and comprise endometrial glands, stroma, blood vessels, and fibrous tissue [188,192]. Although most are benign, malignant transformation has been observed in some cases [192], and estrogen and progesterone play an important role in their pathogenesis, controlling their growth and development [193,194]. Uterine leiomyomas (also called fibroids or myomas) are benign monoclonal neoplasms of the myometrium and represent the most common pelvic tumors in women, affecting more than 70% worldwide [189]. There are three cell populations in fibroids: well-differentiated cells, intermediately differentiated cells, and stem cells, which are believed to be the origin of fibroids [195]. Fibroid-initiating stem cells are more prevalent in women of Afro-American descent and lower in Caucasian women [196]. Searching the relationship between Cu and these uterine diseases revealed few articles on the subject. One study of patients diagnosed with polyps, fibroids, or other benign uterine diseases reported a significant increase in serum Cu levels compared to healthy women [197]. Another showed that Cu levels were higher in patients with uterine fibroids compared to the control group and significantly higher in patients with CC compared to those with fibroids [175]. In patients with endometrial polyps, no differences were found in serum Cu levels compared to the control group [198], but the Cu/Zn ratio was statistically higher, so the authors suggested that oxidative stress would play a role in the pathogenesis of endometrial polyps. By comparing different gynecological diseases, the lowest serum Cu values were found in patients with endometrial polyps and highest in patients with EC, along with elevated Zn levels in uterine fibroids [186].

Endometriosis (EDT) is an estrogen-dependent disease characterized by endometrial-like tissue growing outside the uterine cavity. EDT is considered a chronic and systemic disease, affecting 5–10% of reproductive-age patients in the world [190]. Although EDT is not cancer in itself, it presents similar characteristics: progressive and invasive growth, recurrence, ability to develop its own blood supply, and tendency to metastasize [199]; therefore, it is interesting to know whether Cu has some relationship with this pathology. The first studies reported elevated Cu levels in serum and urine samples from patients with EDT [200,201], which were associated with oxidative stress [200,202]. In patients with advanced-stage EDT [200], a positive correlation was found between Cu and the total oxidant status and between Cu and the oxidative stress index. In another work carried out in animals with induced EDT, the authors demonstrated that elevated Cu levels were positively correlated with the volume of endometriotic-like lesions, high nitrite levels in peritoneal fluid, and increased catalase and glutathione peroxidase activity [202]. In endometriotic lesions, SOD1 has also been found to have increased activity compared to controls [203], which is important for tumor formation [204]. Cu could also stimulate the main signaling pathways of cell proliferation in EDT [205], contributing to malignant transformation within this pathology. Considering that EDT is an estrogen-dependent disease, it is interesting to highlight that Cu is capable of modulating steroidogenesis. It has been observed that, at low levels, this metal can decrease the concentration of estradiol precursor hormones [206], while at high levels, it promotes the expression of enzymes related to the synthesis of this estrogen [207]. We found that the surgical establishment of EDT in mice increased the concentrations of Cu and estradiol, and the administration of a Cu chelating drug decreased both concentrations to values similar to the group with placebo surgery [208]. We also found similar results in another study [209], in which elevated Cu and estradiol levels were efficiently reduced by a Cu chelator in a murine model deficient in tumor necrosis factor (TNF-α) receptor 1 (TNFR1), which presents an aggravated state of EDT [202,210,211].

## 3. Therapeutic Strategies

The recognition that Cu can have a crucial role in disease pathogenesis has led to the development of therapeutic strategies designed to modulate Cu transport and concentrations; to date, the main focus has been on strategies to treat different types of cancer [72,212,213]. Currently, there is significant interest in strategies to mitigate Cu dyshomeostasis, and hence, the attractiveness of applying these therapeutic strategies to the gynecological diseases analyzed in this review. We will focus on the two main strategies, namely Cu chelators, which decrease the bioavailability, and Cu ionophores, which increase intracellular Cu levels. We will subsequently analyze new strategies related to nanotechnology and plant-derived natural compounds, which have been gaining ground as potential treatments for gynecological diseases.

### 3.1. Copper Chelators

A chelator is a chemical compound capable of selectively binding to a particular metal atom or ion through a coordination bond, forming a stable structure [214]. The mechanism of action of Cu chelators involves binding to the metal with subsequent excretion of Cu to inhibit cuproplasia. Historically, Cu chelators were developed to treat Wilson’s disease [66], and the most representative examples are D-penicillamine, trientine, and tetrathiomolybdate (Table 2). While the first two have been used clinically for Wilson’s disease for many years, tetrathiomolybdate is a more recent addition—it has been approved in Europe but is still undergoing clinical trials in the US [65]. Considering the critical role of Cu in cancer progression, different authors investigated whether Cu chelators could serve as an antitumor strategy in animal models and clinical trials [77], promoting the emergence of many reports with interesting results. Cu chelation therapy is promising, not only because of its effectiveness but because these agents have the ability to act selectively on malignant tumors, exerting little toxicity on normal cells [47,79].

#### 3.1.1. D-penicillamine

D-penicillamine is a byproduct derived from penicillin that, in addition to binding Cu with great strength, has the ability to chelate other divalent cations such as Ni, Zn, and Pb [77]. The mechanism of action is based on the chelation of Cu^2+^ ions with the subsequent formation of a stable complex that is excreted through the urine [215]. D-penicillamine is commonly used to treat Wilson’s disease; however, it has been associated with severe toxicity due to numerous adverse effects, such as dystonia, hypersensitivity, pancytopenia, fever, renal failure, congestive heart failure, and tremor, among others [216]. This drug can also be used for cystinuria, rheumatoid arthritis, and heavy metal poisoning [216]. It was shown that Cu chelation by D-penicillamine inhibited neovascularization and human endothelial cell proliferation, affecting angiogenesis [217] and decreasing tumor growth [218]. It also inhibited LOX activity and reduced VEGF expression, causing deficient collagen cross-link formation and delaying tumor progression [219]. In a recent study, the authors observed that treatment with D-penicillamine (but not trientine) caused the inhibition of cell proliferation and EMT by affecting TGF-β/Smad signaling in glioblastoma cells [220]. In oxaliplatin-resistant cervical cancer cells, the combination of D-penicillamine with oxaliplatin or CDDP had a synergistic lethal effect, promoting a greater formation of platinum-DNA adducts, with an increase in the expression of CTR1 and a decrease in ATP7A through the transcription factor Sp1 [221]. Clinical trials with D-penicillamine have been developed for Wilson’s disease, rheumatoid arthritis, cystinuria, and brain tumors.

#### 3.1.2. Trientine

Due to the severe side effects induced by D-penicillamine treatment, triethylenetetramine or trientine was introduced. This drug has a lower Cu-chelating capacity and better tolerability than D-penicillamine and is indicated in those patients with Wilson’s disease who do not tolerate D-penicillamine [222]. Trientine has a polyamine structure that chelates Cu through a stable ring, promoting cupriuresis [222]. The risk of neurological deterioration with trientine is similar to that of D-penicillamine, which usually resolves by reducing the dose [223]. Other adverse events are headache, anemia, arthralgia, rash, and gastrointestinal upset. Trientine has been investigated as a potential anticancer agent. It suppressed tumor development in mice [224] and in hepatocellular carcinoma cell lines [225] and reduced tumor growth in a murine fibrosarcoma model [226]. It also inhibited tumor angiogenesis by decreasing endothelial cell proliferation and expression of CD31 [224] and IL-8 [225]. In addition, trientine is an inhibitor of telomerase [227], an essential factor for cell immortalization that is expressed in most human cancers [228]. Because Cu chelation has been shown to enhance platinum uptake by tumor cells, a small clinical trial combined trientine with carboplatin and pegylated liposomal doxorubicin for the treatment of OC, fallopian tube cancer, and recurrent peritoneal cancer refractory to platinum therapy (ClinicalTrials.gov ID: NCT03480750, Table 3) [229]. The results showed that the combination was safe, but antitumor activity was modest, with no correlation between the clinical response and Cu or Cp levels. This finding was inconclusive, possibly due to the small sample size or the potential influence of ethnic distribution [229].

#### 3.1.3. Tetrathiomolybdate

Another highly specific and widely studied Cu chelator is tetrathiomolybdate, particularly ammonium tetrathiomolybdate (TM), which is rapidly absorbed and has a good safety profile. The first indication of its Cu-binding capacity was the recognition that ruminant animals fed Mo-rich grasses developed a Cu deficiency syndrome (tear disease) [230]. The initial report suggested the administration of molybdates to treat Wilson’s disease; however, a subsequent study showed no clinical benefit in patients [231]. Unlike ruminants, in which rumen cellulose disulfide reacts with Mo, the human gastric mucosa cannot reduce molybdate to the form that can bind Cu [232]. Eventually TM, a reduced form of molybdate, was introduced to diminish Cu levels in humans. If Cu levels are normal, TM is converted to molybdate, incapable of binding Cu, and is excreted via the urine. In the presence of excessive levels of Cu, TM interferes with Cu absorption at the intestinal level when taken with meals and, between meals, forms a stable tripartite complex with serum albumin and circulating Cu to promote biliary excretion, reducing excessive levels of the metal [100,222]. The side effects can be anemia, leukopenia, and increased transaminases, which are easily reversed by reducing the daily dose of TM [233]. Despite the low toxicity of TM, its clinical use is somewhat limited due to the instability of ammonium with oxygen, so a more stable and pharmacologically equivalent TM derivative, bis-choline tetrathiomolybdate (ALXN1840), is also available and is being investigated for the therapy of Wilson’s disease.

Ammonium tetrathiomolybdate has also been shown to reduce tumor growth and function as an effective antiangiogenic agent in both preclinical studies and clinical trials in cancer [98,234,235,236,237,238]. TM can (a) suppress the transcriptional activity of NF-κB, which in turn decreases the expression of angiogenic factors, such as VEGF, FGF, IL-1α, IL-8 [99], (b) induce the degradation of HIF-1α and therefore, reduce the expression of pro-angiogenic factors [97], and (c) suppress Cu chaperone proteins, inhibiting the delivery of Cu to cuproenzymes such as LOX [100,239]. Among these enzymes, inhibition of SOD1 is one of the main therapeutic targets of TM, producing antiangiogenic and antiproliferative effects [240]. Recently, other studies have suggested that TM-induced Cu depletion inhibits MEK1/2 kinase activity, suppressing BRAFV600E-driven tumorigenesis [90,241,242]. In research carried out with OC and EC cell lines, it was found that treatment with TM decreased the protein levels of HIF-1α by mediating its degradation independently of Akt signaling, affecting VEGF levels [97]. It was also observed that trientine or D-penicillamine does not decrease HIF-1α, even at a concentration three times higher than that used with TM [97]. If high Cu levels are reduced, it is possible to sensitize cells to chemotherapy and radiotherapy; therefore, combining these treatments with TM is of interest [237,243,244,245,246]. Research confirmed that Cu depletion sensitized OC cells to therapy with mitomycin C, fenretinide, and 5-fluorouracil by increasing ROS production and inducing DNA damage [237]. TM treatment also improved the efficacy of CDDP in EC and OC cells [245], exerting an antiproliferative effect. TM also enhanced the cytotoxic effects of doxorubicin in EC and OC cells by increasing ROS levels and inducing apoptosis [237,246]. A recent study evaluated the combined effect of TM and lenvatinib (a VEGFR inhibitor) in a model of hepatocellular carcinoma [247]. Tumor burden was positively correlated with Cu concentration, and TM in combination with lenvatinib suppressed tumor growth and angiogenesis to a greater extent than either drug alone, indicating the potential value of this combination as an anticancer treatment.

There is currently no cure for endometriosis, so it is necessary to investigate new treatments that allow the control of EDT progression [248]. Due to the demonstrated implication of Cu in the progression of EDT, our research group first investigated TM as a potential therapy [208,209] and found that it was highly effective in a model with induced EDT. TM decreased the size of the lesions and reduced the elevated levels of Cu and estradiol to physiological levels, along with antiproliferative and antiangiogenic effects [208]. Observing these promising results, we investigated the therapeutic potential of TM in a TNFR1-deficient murine model with induced EDT, which presents an aggravation of the pathology [209]. TM inhibited the EDT progression in the deficient mice, notably affecting cell proliferation, angiogenesis, and oxidative stress while restoring the levels of Cu and estradiol, which are higher in this aggravated version of EDT [209]. TNF-α secretion can be regulated by Cu [212], and several studies have reported the crucial role played by TNF-α, TNFR1, and TNF-α receptor 2 (TNFR2) in EDT [202,210,249,250]. Cell survival, cell proliferation, and cell death occur as a balance between the TNFR1 and TNFR2 signaling pathways, demonstrating the significant crosstalk between them [251,252]. Without TNFR1 expression, TNFR2-dependent pathways that promote tumor progression become relevant [251]. We found that TM also decreased the expression of *Tnfr2* [209], and this is important since blocking TNFR2 has been shown to reduce tumor growth [253] and EDT development [254]. Table 3 shows some of the clinical trials where the effectiveness of Cu chelators in gynecological diseases has been evaluated. As can be observed, despite the promising preclinical results, TM has not yet been investigated as a single or combination therapy in gynecological diseases. The last active clinical trial is a Phase 2 study in breast cancer (ClinicalTrials. gov ID: NCT00195091, Table 3), where it has so far shown that patients with triple-negative breast cancer were more responsive to TM treatment than patients with other breast cancer subtypes [238]. ALXN1840 has only been tested for the therapy of Wilson’s disease (Table 3).

**Table 3:** Examples of clinical trials on drugs related to Cu. Information was obtained from the public database (http://www.clinicaltrials.gov/), accessed on 11 November 2023.

**Disease**	**Trial Phase**	**Intervention**	**Trial ID**	**Status**	Study Completion
Breast Cancer	Phase 2	TM	NCT00195091	Active, not recruiting	06/2025
Wilson’s Disease	Phase 2	ALXN1840	NCT04422431	Completed	05/2023
EOC, TC, PPC	Phase 1–2	Trientine 2HC + PLD + carboplatin	NCT03480750	Completed	12/2019
Advanced cancers	Phase 1	Trientine 4HC + carboplatin	NCT01178112	Completed	08/2014
EOC, TC, PPC	Phase 2	Elesclomol + paclitaxel	NCT00888615	Completed	08/2016
CC	Phase 2	^64^CuII(atsm)	NCT00794339	Terminated	12/2011
CIN	Phase 2	Curcumin	NCT04266275	Not yet recruiting	03/2025
CC	Phase 1–2	Curcumin + radiotherapy	NCT05947513	Not yet recruiting	11/2024
CC	Phase 2	Curcumin	NCT04294836	Withdrawn	12/2023
EDT	Phase 2	Curcumin	NCT04493476	Unknown Status	12/2022
CC, EC	Phase 2	Pembrolizumab + radiation + curcumin + immune modulatory cocktail	NCT03192059	Completed	06/2021
EC	Phase 2	Curcumin	NCT02017353	Completed	10/2016

### 3.2. Copper Ionophores

Unlike the sequestering nature of Cu chelators, Cu ionophores transport this metal into cells, forcing an increase in the intracellular Cu concentration and exerting cytotoxic effects through different pathways [10,255]. Examples of Cu ionophores are disulfiram, clioquinol, elesclomol, and bis(thiosemicarbazone) analogs [mainly CuII(atsm) and CuII(gtsm)]. Several years ago, it was determined that tumor cells were more sensitive to elevated levels of ROS than were normal cells [256]. Despite the promoting effects of Cu on tumor progression, inducing Cu accumulation within cancer cells could promote ROS elevation to take advantage of ROS toxicity as a potential antitumor therapy [257]. Cuproptosis is a specific type of cell death recently postulated by Tsvetkov et al. [258], which is triggered by the accumulation of intracellular Cu. The authors showed the ability of Cu to bind to lipoylated proteins of the tricarboxylic acid (TCA) cycle, promoting increased mitochondrial energy metabolism and toxicity stress, which ultimately causes cell death. The mode of action of Cu ionophores is believed to be interaction with DNA, inhibition of the proteasome, and the ability to displace other metals from the binding sites on critical proteins [259,260].

#### 3.2.1. Disulfiram and Dithiocarbamates

The best-known dithiocarbamates are pyrrolidine dithiocarbamate and diethyldithiocarbamate, the active form of disulfiram (DSF). DSF has been used for many years to treat alcohol dependence since it inhibits the enzyme, aldehyde dehydrogenase (ALDH) [261], and the first evidence of its effectiveness in cancer was in 1977 when it was used in an alcoholic patient with metastatic breast cancer who received DSF and went into spontaneous remission [262]. Since then, its possible use as an anticancer agent has gained interest [263,264,265,266]. DSF has been shown to inhibit cell proliferation, migration, and invasion by altering the nuclear translocation of NF-κB and the expression of *Smad4* [267]. This downregulates proteins such as Snail and Slug, inhibiting EMT and hindering tumor metastasis. In OC cells, DSF also inhibits ALDH [268,269], which has been related to poor prognosis because it promotes resistance to therapy, the maintenance of cancer stem cells, and the mitigation of oxidative stress [270,271]. DSF also prevents the growth of endometriotic lesions by reducing angiogenesis, cell proliferation, and NF-κB expression. In an animal model of endometriosis, DSF increased the serum concentration of malondialdehyde (a marker of lipid peroxidation) and lowered the total antioxidants, TNF-α, and IL-1β compared to the control group [272]. DSF also enhances the anticancer activity of chemotherapeutic drugs, such as CDDP and temozolomide [266,273], which is why DSF is often used in combination therapy. Beneficial effects have been observed in OC, where the combination of DSF with docosahexaenoic acid (DHA) [274] and PARP inhibitors [275] suppressed tumor growth, improving drug sensitivity. In these studies, DSF ameliorated DHA-induced oxidative stress by upregulating Nrf2-mediated *HO-1* (heme oxygenase 1) gene transcription [274] and inhibiting the expression of genes associated with DNA damage repair [275]. In turn, it was demonstrated in chemoresistant OC cells that DSF combined with CDDP synergistically inhibited tumor growth, possibly promoting the downregulation of *Smad3* [276]. By adding Cu, it is possible to enhance the DSF activity in some cases by the DSF/Cu complex formation [266,277,278,279]. Evidence has shown that the main targets of DSF/Cu may be the levels of ROS, the ubiquitin–proteasome system, and NF-κB [263,265,273,280]. DSF/Cu preferentially targets cancer cells and cancer stem cells rather than normal cells [281,282,283,284]. An example of this was observed by Xu et al., where DSF/Cu was cytotoxic in a dose-dependent manner for leukemia stem cells without affecting normal hematopoietic progenitor cells [282]. In another study, DSF increased Cu absorption in cancer cells, with an increase in Cu redox reactions, promoting oxidative stress [285]. In human osteosarcoma cells, DSF/Cu reduced cell growth by autophagy and apoptosis in a ROS-dependent manner with the implication of the ROS/JNK pathway [286], similar to the effects observed in CC cell lines [287]. Although several dithiocarbamates and their derivatives have demonstrated Cu-dependent anticancer activity [288], and promising preclinical results have been observed with DSF, clinical studies in cancer patients have not been successful. When DSF/Cu was administered as monotherapy, it did not produce significant benefits in patients with solid tumors, probably due to insufficient bioavailability of DSF and its metabolite in blood [273].

#### 3.2.2. Clioquinol

The best-known derivative of the 8-hydroxyquinoline class of drugs is clioquinol, which was initially synthesized as an antimicrobial agent for shigellosis and intestinal amebiasis [289]. It has subsequently been studied in different diseases ranging from neurodegenerative disorders to cancer [280]. The first study to evaluate clioquinol as an antitumor agent showed that it decreased viability by inducing apoptosis in eight different cancer cell lines and prevented the growth of OC xenografts in mice [290], with similar results in prostate cancer cells and xenografts [291]. Another study reported that a different OC cell line was sensitive to the combination of clioquinol and DHA, with toxicity mediated by the action of PPARα [292]. Similar to DSF, the anticancer activity of clioquinol is enhanced by Cu and has been linked to proteasome inhibition and oxidative stress [290,293,294,295]. One of the targets of clioquinol is the X-linked inhibitor of an apoptosis protein (XIAP), which modulates caspase activity, allowing selective action with apoptosis being only triggered in cancer cells [293] and an insignificant effect in normal cells. Clioquinol increases the tissue content of Cu^2+^, indicating that the clioquinol–Cu^2+^ complex could be the metabolite that triggers the death of cancer cells, and it could be formed intracellularly or extracellularly and transported into the cells [296]. Clioquinol can trigger autophagy by inducing LC3 lipidation and autophagosome formation in myeloma and leukemia cells [295]. It can also exacerbate the anticancer activity of macrophages toward CC cells, promoting the secretion of interleukins and cytokines, such as TNF-α [297]. Although clioquinol has shown selective promise in cancer chemotherapy, it has also caused serious neurotoxicity that led to its clinical prohibition [298]. Various routes of administration or combination with other drugs for safer application are still being investigated [255,299]. Further derivatives of 8-hydroxyquinoline, such as PBT2 and nitroxoline, might have greater effectiveness as anticancer agents by inhibiting the proliferation of cancer cells with fewer side effects [300,301]; nevertheless, they have not yet been tested in gynecological diseases.

#### 3.2.3. Elesclomol and Derivatives

Elesclomol is a carbohydrazide, bis(thio-hydrazide amide), developed from a parent molecule, which had anticancer activity but was chemically and metabolically unstable [302]. Elesclomol is stable and causes a 10-fold increase in cancer cell cytotoxicity compared to the parent molecule. It induces oxidative stress in cancer cells [303,304,305,306] and alters mitochondrial metabolism, particularly the TCA cycle, promoting cuproptosis [258,307]. The anticancer activity is due to the formation of an elesclomol–Cu^2+^ complex [308] that facilitates transport into the mitochondria, where reduction to Cu^+^ leads to oxidative stress and subsequent cell death [305]. Using CRISPR-Cas9 deletion, the mitochondrial protein ferredoxin-1 (FDX1) was shown to bind to the elesclomol–Cu^2+^ complex, reducing Cu^2+^ to Cu^+^ and promoting the anticancer activity of this ionophore [307]. In a mouse model, treatment with elesclomol–Cu^2+^ inhibited the development of endometriosis through FDX1-mediated cuproptosis [309].

Inactivating mutations in the AT-rich interactive domain-containing protein 1A (ARID1A) are found more frequently in gynecological cancers [310], and in 14 gynecological cancer cell lines, loss of ARID1A caused increased levels of ROS. Elesclomol inhibited tumor growth and induced apoptosis in these ARID1A mutant cells [303]. In another in vitro study, elesclomol with anisomycin inhibited the proliferation of OC stem cells, while elesclomol alone was ineffective [311]. In an OC relapse model, both disulfiram and elesclomol promoted cell death following treatment with carboplatin compared to carboplatin alone [312]. Although these laboratory studies have been promising, when elesclomol was administered in clinical trials as monotherapy or in combination with other chemotherapeutics for different types of tumors [313,314,315], the benefit has been small or negligible. In a phase II clinical study, elesclomol with paclitaxel was used as a treatment for cisplatin-resistant OC, fallopian tube cancer, and peritoneal cancer [313]. Although this combination showed a good safety profile, it did not produce the expected response, possibly because elesclomol is not effective at elevated levels of the enzyme, lactate dehydrogenase (LDH) [313,316], suggesting that elesclomol may be less effective in situations with a high rate of glycolysis. Hypoxia has been associated with more aggressive tumors that have elevated LDH levels. Elesclomol is more effective in non-hypoxic conditions because it interferes with metabolic processes in oxygenated tumor cells [305]. For more information, in a recent review, special attention is paid to elesclomol as an anticancer therapy [317].

#### 3.2.4. Bis(thiosemicarbazones)

Thiosemicarbazones and bis(thiosemicarbazones) are capable of binding to metals, forming stable, lipophilic, and often neutral complexes [318]. Diacetyl-bis-(N4-methylthiosemicarbazonato)-copper(II) [CuII(atsm)] and glyoxal-bis-(N4-methylthiosemicarbazonato)-copper(II) [CuII(gtsm)] have a similar structure, but differences in their redox behavior [319]. Due to the elevated Cu levels in cancer, these ionophoric Cu compounds have been investigated to determine if they could selectively treat tumor cells without altering normal cells. A study on a TRAMP (transgenic adenocarcinoma of the mouse prostate) model documented CuII(gtsm) selectivity for cancer cells with high Cu levels [260]. CuII(gtsm) increased ROS in TRAMP cells along with decreased GSH but did not do so in normal mouse prostate epithelial cells. In another study investigating CuII(atsm) and CuII(gtsm) as anticancer agents [320], CuII(gtsm) was cytotoxic against prostate cancer cells and significantly reduced the tumor burden, while the CuII(atsm) action was insignificant. It is important to note that CuII(gtsm) dissociates upon entering the cell, increasing the intracellular bioavailability of Cu and causing toxicity, while the ligand (H2gtsm) is recycled out of the cell and re-enters with more re-coordinated Cu [320]. This property explains how increasing extracellular Cu improves the anticancer activity of CuII(gtsm), which could be applicable in patients with elevated serum Cu levels. In contrast, CuII(atsm) retains Cu due to its lower reduction potential in intracellular reducing environments [318,321]. CuII(atsm) is selective toward cells with low oxygen levels since a more forced-reducing environment (such as hypoxia) leads to the reduction of CuII(atsm) and its dissociation [319,322], as demonstrated in hypoxic neuroblastoma cells where CuII(atsm) caused higher intracellular Cu levels compared to control cells [321]. As a result of this characteristic, radiolabeled Cu complexes have been synthesized that are theranostic, i.e., they allow simultaneous imaging diagnosis and therapy [323], especially with the ^64^Cu isotope [324]. In one of the first studies, ^64^CuII(atsm) demonstrated anticancer activity as a radiotherapy agent in a hamster colon cancer model, increasing survival time without toxic effects [325]. Several studies in cancer patients have been performed to evaluate survival concerning the uptake of these isotopes. When ^60^CuII(atsm) was used as a marker of hypoxia, higher uptake predicted a worse prognosis in patients with CC [326]. In another study of CC, ^60^CuII(atsm) promoted the overexpression of VEGF, cyclooxygenase-2, epidermal growth factor receptor (EGFR), carbonic anhydrase 9 (CA-9), along with an increase in cell death [327]. In a comparative study, ^64^CuII(atsm) was shown to be more effective than ^60^CuII(atsm) in obtaining better-quality images for patients with CC [328]. Cu ionophores may offer great selectivity toward cancer cells with antitumor activity against different cancer types but, to date, most preclinical results have not been replicated in patient trials, reflecting the need to better understand the action mechanism and pharmacokinetics of these compounds [47].

### 3.3. New Therapeutic Strategies

Recognizing that alterations in Cu homeostasis are involved in the pathogenesis of various diseases and the potential value of Cu-based therapies has prompted the development of new compounds based on Cu [329]. Nanotechnological strategies and natural plant-derived compounds have been gaining ground as potential treatments for gynecological diseases.

#### 3.3.1. Cu-Based Nanoparticles

Nano-oncology involves the use of nanotechnological strategies for cancer treatment. In this sense, nanoparticles (NPs) can function directly as an antitumor treatment or as a vehicle to mediate the controlled administration of drugs to increase their effectiveness and decrease their side effects [330]. Cu-based NPs (CuNPs) form a stable structure with a diameter of 10–50 nanometers, and they are used in a variety of industrial processes that release them into the environment. CuNPs pass through wastewater treatment plants into water systems and enter vegetation through the agricultural use of fertilizers and pesticides [331], but current levels of environmental exposure have not been linked to disease pathogenesis. Due to their high surface-to-volume ratio, CuNPs can interact efficiently with tissues, an attractive characteristic for use in oncology. The Cu-induced toxicity of CuNPs is related to oxidative damage through increasing ROS, the formation of peroxy radicals, lipid peroxidation, and reduction in CCO activity [331]. The production of NPs can be accomplished via ‘green synthesis’ using plants, algae, and microorganisms, which is presumably an environmentally friendly process.

Copper-based nanoparticles have been investigated as antitumor agents in several types of cancer [332,333,334,335]. In OC cell lines, CuNPs synthesized from a *Camellia sinensis* leaf extract were effective in causing tumor cell death [332]. In CC lines, CuNPs synthesized from a pumpkin seed extract caused a decrease in cell viability, increased production of ROS, apoptosis induction, and the suppression of cell migration with the antitumor effect linked to inhibition of the PI3K/Akt signaling pathway [335]. Similarly, other CuNPs synthesized using an extract of *Houttuynia cordata* were effective as antitumor agents in CC cells [336]. The development of CuNP–transferrin loaded with doxorubicin also successfully inhibited tumor growth in mice [337], and these NPs were able to specifically enter CC and breast cancer cell lines that overexpressed the transferrin receptor. CuNPs synthesized from the red alga *Pterocladia capillacea* and loaded with nedaplatin improved the antitumor activity in OC compared to treatment with nedaplatin alone [338]. There have recently been reports on an innovative and promising strategy to improve the precision of cancer treatment by using NPs in photothermal therapy. Copper sulfide (CuS) NPs target tumor cells and enter the nucleus; subsequent near-infrared laser irradiation activates the NPs to increase the temperature within the nucleus, leading to apoptosis of the tumor cell. The main goal is to target both the primary tumor and malignant cells that have escaped, thereby minimizing metastasis. Initial studies in mice have demonstrated that photothermal therapy was effective and safe in eliminating residual CC cells and preventing tumor recurrence [339]. Another investigation showed that CuS NPs, together with laser irradiation, effectively killed tumor cells in mouse models of OC with a minimal effect on surrounding healthy tissue [340].

#### 3.3.2. Natural Compounds Derived from Plants

In recent years, attempts have been made to identify natural molecules that can be used in oncology. Although these compounds are considered to act as antioxidants, the objective is to have them work as pro-oxidants in the presence of Cu, catalyzing ROS formation and DNA degradation. In this regard, several plant-derived Cu-binding molecules have been reported to exert anticancer effects and increase the antitumor activity of other known chemotherapeutics with low side effects.

##### Curcumin

Curcumin, a bioactive turmeric polyphenol derived from the rhizomes of *Curcuma longa*, chelates Cu with a wide range of biological effects, including antioxidant, anti-inflammatory, and antimicrobial properties when examined in a variety of laboratory models [341]. It may also have protective effects against different types of cancer, including lung cancer, breast cancer, colon cancer, and gynecological cancers [342,343,344], but when given orally, it is poorly absorbed and rapidly inactivated, limiting the potential for clinical use. Strategies to improve the pharmacokinetics have included the creation of curcumin-metal NP, and a curcumin–Cu complex was shown to have higher anticancer activity compared to curcumin alone [345,346]. In an in vitro study with EC cells, curcumin treatment suppressed tumor growth, inhibited cell proliferation, and promoted ROS-induced apoptosis [347]. It also attenuated cell migration by increasing the expression of the Slit2 protein, causing the downregulation of SDF-1 (stromal cell-derived factor 1) and CXCR4 (C-X-C motif chemokine receptor 4) and, therefore, of MMP (matrix metalloproteinase) 2 and 9. The decrease in the expression of MMPs, with implications for invasion, migration [348], and cell proliferation [349], has been documented by other investigators. In CC cells, curcumin suppresses proliferation and invasion by affecting the Wnt/β-catenin and NF-κB pathways [350] and elevates intracellular ROS levels but not in healthy epithelial cells, leading to cell-specific apoptosis [351]. Regarding OC, curcumin has shown great anticancer potential because it suppresses cell cycle progression, promotes apoptosis and autophagy, and inhibits tumor metastasis, so current efforts are focused on finding suitable derivatives to overcome the pharmacokinetic limitations (reviewed in detail by Liu et al. [352]).

Curcumin has also been studied in animal models of PCOS, where it (a) reduces testosterone levels and increases estrogen levels [353], (b) promotes an anti-inflammatory mechanism by reducing proteins involved [354], (c) improves ovarian function [354,355], (d) improves the levels of total cholesterol, HDL, LDL, and triglycerides [353,355], and (e) decreases malondialdehyde levels and increases the activities of SOD, catalase, and GSH [353,355], among other effects. However, the results in clinical trials are discrepant, probably due to the inclusion and exclusion criteria used and the number of participants. For example, in a clinical trial in patients with PCOS, curcumin reduced serum insulin, fasting glucose, and the index of insulin resistance (HOMA-IR) [356], while no differences were found in another clinical trial [357]. Regarding EDT, in an in vitro study, curcumin induced a lower expression of ICAM-1, VCAM-1, IL-6, IL-8, and MCP-1 by inhibiting the activation of NF-κB induced by TNF-α without affecting the viability of endometriotic stromal cells [358]. However, another study showed that the number of endometriotic lesions, their volume, and the degree of adhesions, along with the levels of IL-1β, IL-6, HIF-1α, and VEGF, were reduced in mice treated with curcumin compared with the control group [359]. Reduced secretion of pro-angiogenic chemokines and pro-inflammatory cytokines, upregulation of IL-10 and IL-12, and abrogation of IKKα/β, NF-κB, STAT3, and JNK signaling pathways have been demonstrated in eutopic endometrial stromal cells from patients with EDT treated with curcumin [360]. Curcumin reduces cell survival, VEGF expression, and cell proliferation in endometriotic cells [361] and lowers estradiol levels that are elevated in EDT [362]. In addition, curcumin may decrease EDT by promoting apoptosis through p53-dependent and -independent mitochondrial pathways [363]. Patients with EDT receiving a combination of quercetin, turmeric, and N-acetylcysteine reported a reduction in pain and lower use of non-steroidal anti-inflammatory drugs (NSAIDs) [364]. The role of curcumin and other plant-derived compounds as potential treatments for EDT has been reviewed in detail by Meresman et al. [365]. Table 3 shows some clinical trials that use or have used curcumin alone or with other treatments.

##### Coumarins

Coumarins are found in plants, such as *Rutaceae* and *Umbelliferae,* and belong to the benzo-α-pyrone family. These compounds have anti-inflammatory, antioxidant, and antitumor activities [366]. A coumarin–Cu complex [367,368] and a coumarin–Cu–thiosemicarbazone hybrid have been effective antiproliferative agents in cell lines of different types of cancer [369], and a coumarin–amide–Cu complex was shown to have greater antitumor capacity than CDDP in a breast cancer cell line [370]. Two studies in OC cells have demonstrated the antitumor effect of two natural derivatives of coumarin, 4-methylumbelliferone, and Osthole. These compounds reduce cell proliferation by affecting the PI3K/Akt and MAPK pathways [371] or induce several cell death mechanisms [372]. Osthole has also been tested in CC cells, where it reduces cell viability, proliferation, migration, and invasion, along with inducing apoptosis [373]. The combination of Osthole with CDDP reduced cell proliferation and enhanced apoptosis in CC cells to a greater extent than CDDP alone, notably downregulating the PI3K/Akt pathway [374]. Other coumarin derivatives have similar effects on this pathway in CC cells [375,376]. Imperatorin, a furanocoumarin derivative, was effective in an animal model of EDT [377], significantly inhibiting the growth of ectopic endometrium, improving the histopathological characteristics, and inhibiting the PI3K/Akt/NF-κB pathway. Auraptene, a coumarin derivative found in citrus fruits, decreased the inflammation and elevated the fertilization rate in isolated oocytes in a mouse model of PCOS [378]. The drug lowered ROS levels and elevated intracellular GSH levels, indicating that auraptene could be a potential candidate to improve oocyte maturation and fertilization capacity in patients with PCOS [378]. This has subsequently been confirmed in a mouse model of in vitro fertilization and early embryo development [379].

## 4. Concluding Remarks

Gynecological diseases are characterized by high prevalence, morbidity, and mortality and it is essential to investigate the pathogenesis and possible diagnostic and therapeutic strategies for these disorders. Over the years, the importance of Cu in health and disease has been increasingly recognized, and research on Cu has gained prominence, with extensive efforts to document and understand its complex roles and diverse mechanisms of action. Although Cu is crucial for many physiological functions, it is also potentially toxic at altered levels, and specific regulatory mechanisms normally prevent Cu dyshomeostasis. Documenting these mechanisms and the alterations that can occur has revealed that Cu has a critical role in the pathogenesis of several diseases, particularly cancer, where Cu is involved in every step from tumorigenesis to metastasis. With time, different therapeutic options based on Cu have emerged for a variety of disorders with promising results, both in animal models and in clinical trials. Some strategies are based on reducing high levels of Cu with chelators to slow the progression of specific gynecological diseases. Other drugs, such as Cu ionophores, can force Cu into cells to take full advantage of its toxic role and induce tumor cell death. Notably, these two categories of drugs mediate opposite actions: Cu chelators inhibit cuproplasia, while Cu ionophores induce cuproptosis. Given the attractiveness of altering Cu levels as a therapeutic strategy, the need to continue investigating these types of drugs is evident, and this will also require further understanding of the pathogenesis of each disorder and the potential role of Cu dyshomeostasis. Ongoing research is an essential stage in the discovery of more effective treatments to target specific genes and influence distinct signaling pathways. The development of new Cu-based compounds holds great promise to revolutionize diagnostic and therapeutic strategies, especially for those gynecologic diseases with high mortality.

## Figures and Tables

**Table 1:** Functions of the main cuproenzymes.

Cuproenzyme	Function
LOX	Required for the formation of the extracellular matrix.
SOD	Catalyzes the conversion of superoxide radicals to molecular oxygen and hydrogen peroxide.
Cp	Multicopper ferroxidase; principal Cu carrier in serum.
Hephaestin	Multicopper ferroxidase. It supports the transportation of Fe released from intestinal enterocytes.
CCO	Electron transfer protein. It catalyzes ATP production.
Tyrosinase	Catalyzes phenol oxidation; it is required for melanin synthesis, a fundamental pigment for hair, skin, and eyes.
DβH	Oxidoreductase. It catalyzes the conversion of dopamine to epinephrine.
MEK	Kinases that belong to the mitogen-activated protein kinase cascade and that mainly promote cell proliferation and survival.
ULK1/2	Autophagy-initiating kinases.
MEMO1	Regulation of cell motility and ROS production.

**Table 2:** Examples of Cu chelators.

**Compound Name**	**Chemical Formula**	**Structural Formula ^1^**
D-penicillamine	C_5_H_11_NO_2_S	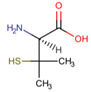
Trientine	C_6_H_18_N_4_	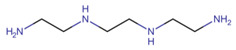
Tetrathiomolybdate	MoS_4_	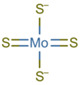

^1^ Structural formulas were obtained from the DrugBank public database (http://www.drugbank.com/), accessed on 11 November 2023.

## Abbreviations

Akt	Protein kinase B
ALDH	Aldehyde dehydrogenase
ALXN1840	Bis-choline tetrathiomolybdate
ARID1A	AT-rich interactive domain-containing protein 1A
Arp	Actin-related proteins
ATOX1	Antioxidant chaperone 1
ATP	Adenosine triphosphate
ATP7A	Copper-transporting ATPase alpha
ATP7B	Copper-transporting ATPase beta
BRAF	Serine/threonine-protein kinase B-raf
CA-9	Carbonic anhydrase 9
Cas9	CRISPR-associated protein 9
CC	Cervical cancer
CCDC	Coiled-coil domain containing protein
CCO	Cytochrome C oxidase
CCS	Copper chaperone for superoxide dismutase
CD31	Cluster of differentiation 31
CDDP	Cisplatin
CIN	Cervical intraepithelial neoplasia
CMRGs	Copper-metabolism related genes
COMMD	Copper metabolism MURR1 domain-containing protein
COX11	CCO copper chaperone 11
COX17	CCO copper chaperone 17
COX19	CCO assembly factor 19
Cp	Ceruloplasmin
CRISPR	Clustered regularly interspaced short palindromic repeats
CTR1	Copper transporter 1
CTR2	Copper transporter 2
Cu	Copper
Cu IUDs	Copper intrauterine devices
CuS NPs	Copper sulfide nanoparticles
CXCR	C-X-C motif chemokine receptor
DβH	Dopamine-β-hydroxylase
DCYTB	Duodenal cytochrome B
DHA	Docosahexaenoic acid
DMT1	Divalent metal transporter 1
DSF	Disulfiram
EC	Endometrial cancer
ECM	Extracellular matrix
EDT	Endometriosis
EGFR	Epidermal growth factor receptor
EMT	Epithelial-mesenchymal transition
EOC	Epithelial ovarian cancer
ERK	Extracellular signal-regulated kinase
FDX1	Ferredoxin-1
FGF	Fibroblast growth factor
GSH	Glutathione
2HC	Dihydrochloride
4HC	Tetrahydrochloride
HIF-1α	Hypoxia-inducible factor 1-alpha
HO-1	Heme oxygenase 1
HOMA-IR	Homeostatic model assessment for insulin resistance
HPV	Human papillomavirus
HREs	Hypoxia response elements
ICAM	Intercellular adhesion molecule
IKKs	Inhibitory kappa B kinases
IL	Interleukin
IMS	Mitochondrial intermembrane space
IR	Insulin resistance
JNK	c-Jun N-terminal kinase
LC3	Microtubule-associated protein light chain 3
LDH	Lactate dehydrogenase
LOX	Lysyl oxidase
LOXL	LOX-like proteins
MAPK	Mitogen-activated protein kinase
MCP-1	Monocyte chemoattractant protein-1
MEK	Mitogen-activated protein kinase kinase
MEMO1	Mediator of cell motility 1
MMP	Matrix metalloproteinase
MT	Metallothionein
MTF1	Metal-regulatory transcription factor 1
NADPH	Nicotinamide adenine dinucleotide phosphate
NF-κB	Nuclear factor kappa B
NO	Nitric oxide
NPs	Nanoparticles
Nrf2	Nuclear factor erythroid 2-related factor 2
NSAID	Non-steroidal anti-inflammatory drug
OC	Ovarian cancer
p53	Tumor protein p53
PARP	Poly (ADP-ribose) polymerase
PBT2	5,7-dichloro-2-[(dimethylamino)methyl]-8-hydroxyquinoline
PCOS	Polycystic ovary syndrome
PDGF	Platelet-derived growth factor
PI3K	Phosphoinositide 3-kinase
PLD	Pegylated liposomal doxorubicin
PPAR	Peroxisome proliferator-activated receptor
PPC	Primary peritoneal cancer
RAF	Rapidly accelerated fibrosarcoma
ROS	Reactive oxygen species
SCO1	Synthesis of cytochrome C oxidase 1
SDF-1	Stromal cell-derived factor 1
SOD	Superoxide dismutase
STAT	Signal transducer and activator of transcription
STEAP	Six-transmembrane epithelial antigen of the prostate
TC	Fallopian tube cancer
TCA	Tricarboxylic acid
TGF-β	Transforming growth factor beta
TGN	Trans-Golgi network
TM	Ammonium tetrathiomolybdate
TMD	Transmembrane domain
TNF	Tumor necrosis factor
TNFR	TNF receptor
TRAMP	Transgenic adenocarcinoma of the mouse prostate
ULK	Unc-51 like autophagy activating kinase
VCAM	Vascular cell adhesion protein
VEGF	Vascular endothelial growth factor
VEGFR	VEGF receptor
WASH	Wiskott–Aldrich syndrome protein and SCAR homolog
XIAP	X-linked inhibitor of apoptosis protein
